# Merging into the mainstream: the evolution of the role of point-of-care musculoskeletal ultrasound in hemophilia

**DOI:** 10.12688/f1000research.16039.1

**Published:** 2019-07-09

**Authors:** Nihal Bakeer, Amy D Shapiro

**Affiliations:** 1 Indiana Hemophilia and Thrombosis Center, 8326 Naab Road, Indianapolis, IN, 46260, USA

**Keywords:** Hemophilic arthropathy, joint disease, hemophilia, musculoskeletal ultrasound, point-of-care ultrasound, hemarthrosis, synovitis

## Abstract

Bleeding with resultant hemophilic arthropathy constitutes the largest cause of morbidity in patients with hemophilia. It results from repeated bleeding episodes in the joint and is characterized by synovial hypertrophy and cartilage and bony destruction. Hemophilic arthropathy assessment is a continually evolving process and is particularly challenging in children and young adults in whom joint disease may be missed or underestimated as obtaining serial “baseline” magnetic resonance imaging scans of multiple clinically asymptomatic or nearly asymptomatic joints may be unjustifiable and cost-ineffective. Musculoskeletal ultrasound—particularly, point-of-care musculoskeletal ultrasound—has emerged as a promising imaging modality for the early detection and management of hemophilic arthropathy, and for the evaluation of hemarthrosis and painful musculoskeletal episodes in patients with hemophilia. This review summarizes currently available data on the emerging role of this new imaging modality, its limitations, and gaps in knowledge. The review also raises unanswered questions, highlights the need for consolidated research efforts, and delineates future directions expected to advance this technology and optimize its use in this patient population.

## Introduction

Hemophilia A and B are X-linked congenital bleeding disorders characterized by reduced or absent levels of coagulation factors VIII or IX, respectively. The disease is classified, on the basis of residual coagulation factor activity level, as severe (<1%), moderate (1–5%), or mild (5–40%). Spontaneous hemarthrosis is the hallmark of severe hemophilia, and hemophilic arthropathy (HA), characterized by synovial hypertrophy and cartilage and bony destruction, which ultimately results from repeated joint bleeding episodes ^1^. The musculoskeletal consequences of hemophilia constitute the largest cause of morbidity in persons with hemophilia (PwH) ^2^; musculoskeletal disease may be minimized by early initiation of prophylaxis yet has continued to occur ^3– 9^, and a single episode of hemarthrosis can impact long-term joint outcomes ^10, 11^. There is evidence that the key processes involved in the pathogenesis of HA include intra-articular inflammation, mediated by hemoglobin release, iron deposition, cytokines, hydroxyl radicals, and the “inflammasome”, and neo-angiogenesis, mediated by pro-angiogenic factors such as vascular endothelial growth factor ^12, 13^. However, the pathophysiology of HA remains incompletely understood, and the pathways are yet to be fully elucidated. HA remains a critical focus of research and study as this debilitating disease negatively impacts quality of life, physical activity, and bone health of the aging PwH population, whose life span is now comparable to that of the normal population ^3, 4, 14– 16^. A gap continues to exist in our ability to detect and impact this sequela at the preclinical or asymptomatic phase when the disease process is early and potentially reversible.

## Evaluation of hemophilic arthropathy

Evaluation of HA is a dynamic, multi-dimensional process which employs clinical outcome assessment tools, including the World Federation of Hemophilia Physical Examination Score (Gilbert score), the hemophilia joint health score (HJHS) ^17– 19^, and a number of imaging modalities ^20, 21^. X-ray grading systems/scores include the Arnold–Hilgartner score (progressive, soft tissue assessment included) ^22^ and the World Federation of Hemophilia–recommended and widely used Pettersson score (additive, soft tissue excluded) ^23^. X-rays are widely available and may capture advanced joint changes but are insensitive to early change and unreliable for cartilage and soft tissue evaluation ^1^. These limitations necessitated the development of a number of HA scoring systems using joint magnetic resonance imaging (MRI) ^24– 26^. The most widely used, developed in 2012 by the International Prophylactic Study Group (IPSG), merges two scales of a combined MRI scoring scheme into a single scale ^27^. This scale, which includes soft tissue and osteochondral subscores, proved useful for the analysis of both early and moderate stages of arthropathy, comparison among different studies and patient populations, and the evaluation of prophylactic factor infusion regimens. To date, joint MRI remains the gold standard for the evaluation of HA, providing a comprehensive multi-tissue evaluation with sensitivity to detect early joint pathology and changes over time ^26– 29^. However, MRI is time-consuming, expensive, not consistently readily accessible, and may require sedation in young children. MRI also detects a large number of minor changes over time, not all of which are necessarily clinically meaningful, and their impact on joint outcomes has yet to be determined ^29, 30^. Therefore, obtaining serial “baseline” MRI scans of multiple clinically asymptomatic or nearly asymptomatic joints of children and young adults, may be unjustifiable and rather cost-ineffective. Conversely, if joint evaluation is restricted to clinically manifest joint disease, early arthropathic changes in this patient population will be missed without consistent sensitive monitoring.

## Musculoskeletal ultrasound in hemophilia

It is not surprising—given its accessibility, wide availability, safety, efficiency, low cost of examination, and lack of interference of susceptibility artifacts on gradient-echo MRI sequences in joints with hemosiderin deposition, including the “blooming” effect ^28^—that musculoskeletal ultrasound (MSKUS), particularly point-of-care MSKUS (POC-MSKUS), has emerged as a promising imaging modality for the early detection and management of HA ^19, 30– 36^ and for the evaluation of hemarthrosis and painful musculoskeletal episodes in PwH ^37– 40^. MSKUS can provide a detailed and dynamic assessment of synovial joints and periarticular structures, including tendons, ligaments, and muscles, using high-resolution, high-frequency transducers (3.5–15 MHz) with power Doppler (PD) imaging. As this imaging modality gained attention, so did the debate and controversy about its reliability, diagnostic accuracy, and ultimately impact on joint outcomes in PwH ^1, 33, 41, 42^, but an important distinction to make prior to critically reviewing the role of MSKUS in the evaluation of HA is that between full-joint MSKUS and POC-MSKUS. Full-joint MSKUS is a 360° assessment of the joint, using all standard scanning planes, and is performed by expert radiologists and ultrasonographers. POC-MSKUS is a focused evaluation of the joint, using limited scanning planes or an abbreviated scanning protocol, and is usually carried out by trained clinicians at the patient’s bedside to answer specific yes–no questions about the status of the joint.

Compared with conventional MRI in the evaluation of hemarthrosis, MSKUS was found to be extremely sensitive in detecting low concentrations of intra-articular blood (as low as 5%) and in discriminating between bloody and non-bloody (serous/simple) fluid ^43^, making it useful in determining the etiology of acute painful musculoskeletal episodes in PwH as arthritis-mediated or bleed-related ^37, 38^. Radiographic biomarkers of HA include both soft tissue changes and osteochondral abnormalities, both of which can be detected with variable accuracy by MSKUS ^32, 33, 42, 44, 45^. In 2014, in an observational descriptive study, Sierra Aisa *et al*. showed that MSKUS is useful in detecting joint bleeds, synovial hyperplasia, and joint erosions, and the results were comparable to those of joint MRI, but MRI better detected bone cysts and cartilage loss ^42^. Similarly, in 2015, Doria *et al*. conducted a cross-sectional study to assess the reliability of interpretation of MSKUS findings according to data blinding in maturing hemophilic joints (knees and ankles) and to determine the diagnostic accuracy of MSKUS compared with MRI for assessing joint components ^41^. The authors concluded that, if performed by experienced radiologists using a standardized protocol, MSKUS is highly reliable to assess soft tissue abnormalities (synovial hypertrophy and hemosiderin deposition) in ankles and knees, and substantially to highly reliable to assess osteochondral changes in these joints, with high sensitivity to diagnose erosions and cartilage loss, regardless of the severity of arthropathy, and poor sensitivity and negative predictive values for depicting subchondral cysts in advanced disease.

Unique to MSKUS, PD imaging allows the evaluation of synovitis by measuring synovial thickness and hypervascularity, which correlates well with the significantly more expensive dynamic contrast–enhanced MRI in hemophilic joints ^13, 46^. PD imaging is valuable as vascular remodeling and angiogenesis are known to contribute significantly to bleed propagation and development of HA as shown in human and mouse studies ^46– 50^. There is evidence that increased PD signals at baseline correlate with acute pain and more bleeding episodes and that PD signals significantly increase at the time of acute bleeding ^38^.

The most comprehensive systematic review and semi-quantitative assessment of currently available evidence on the value of MSKUS in the assessment and management of HA in children and adults with hemophilia and von Willebrand disease were provided by Ligocki *et al*. in 2017 ^51^. The review included 20 articles investigating either the diagnostic accuracy of MSKUS and/or MSKUS scanning protocols and scoring systems for the assessment of HA. Two independent reviewers evaluated 14 retrospective cross-sectional studies examining the diagnostic accuracy of MSKUS in the assessment of HA in children and adults ^10, 37, 38, 41, 42, 44, 46, 52– 58^, both for reporting quality using the Standards for Reporting of Diagnostic Accuracy (STARD) tool and for methodological quality using the Quality Assessment of Diagnostic Accuracy Studies 2 (QUADAS-2) tool. With STARD, 1 out of 14 and 8 out of 14 studies were scored to be of high and moderate reporting quality, respectively. Assessment with QUADAS-2 reported 2 out of 14 and 6 out of 14 studies as having high and moderate methodological quality, respectively. The remainder of the studies were scored as low or very low. These studies were also assessed for outcome measures from three clinimetric constructs (construct validity, criterion validity, and reliability) as follows: (1) clinical joint assessment tools: Gilbert and IPSG scores ^17, 24, 59^; (2) radiographic scores: Arnold and Hilgartner ^22^ and Pettersson ^23^; and (3) MSKUS measures: Muça-Perja *et al*. progressive score ^31^ and five other additive scores by Klukowska *et al*. ^53^, Querol *et al*. ^60^, Melchiorre *et al*. ^10^, Martinoli *et al*. ^45^, and Doria *et al*. ^41^.

Six studies that described MSKUS scoring systems ^10, 31, 41, 45, 53, 60^ were included in the review and the authors divided the parameters of the ultrasound scoring systems into soft tissue changes (hemarthrosis/effusion, hemosiderin deposition, synovial hypertrophy and/or reaction and fibrotic septa) and osteochondral abnormalities (bone erosion, osteophytes, subchondral cysts, bone remodeling, and cartilage damage). The only parameter proposed in all scoring systems was synovial hypertrophy.

Five grey-scale ultrasound scanning protocols were included in this review ^45, 60– 63^. With the exception of Martinoli’s POC protocol ^64^, PD evaluation of soft tissue vascularity was suggested by all remaining full-joint diagnostic protocols. The only other POC protocol evaluated in this review was that of Ceponis *et al*. ^38^. The protocols of both Martinoli *et al*. and Ceponis *et al*. aim to simplify the scanning technique for elbows, ankles, and knees so they can be applied by clinical providers at the bedside without requiring radiologists or radiology technicians. Items included in a number of MSKUS scanning protocols and scoring systems used in the assessment of HA are shown in Table 1 ^33^. The score proposed by Doria *et al*. ^41^, not shown in the table but included in the systematic review, is an ultrasound-based imaging scale with an additive score (0–14) that was adjusted to the subscores of the IPSG MRI scale (0–17) ^27^. To enable correspondence between ultrasound and IPSG MRI scores, items that are not technically feasible by ultrasound, including surface erosions, subchondral cysts, and full-thickness loss of joint cartilage in at least half of the joint surface were excluded ^41, 65^.

**Table 1.** Items included in different scanning protocols and scoring systems for ultrasound assessment of hemophilic arthropathy.

Authors	Effusion (synovial fluid or hemarthrosis)	Synovial hypertrophy	Synovial hyperemia	Hemosiderin deposition	Cartilage abnormalities (cartilage loss, hyperechogenicity, thinning)	Bone abnormalities (erosion, subchondral cysts, osteophytes)	Evaluated joints
Klukowska *et al*. ^53^ (2001)	Yes	Yes	Yes	No	Yes	Yes	Knee
Zukotynski *et al*. ^61^ (2007)	Yes	Yes	No	Yes	Yes	Yes	Knee, ankle
Melchiorre *et al*. ^10^ (2011)	Yes	Yes	No	Yes	Yes	Yes	Elbow, knee, ankle
Muça-Perja *et al*. ^31^ (2012)	No	Yes	Yes	No	Yes	Yes	Knee, ankle
Martinoli *et al*. ^45^ (2013)	Yes ^a^	Yes	No	No	Yes	Yes	Elbow, knee, ankle
Kidder *et al*. ^37^ (2015)	No	Yes	Yes	No	Yes	Yes	Elbow, knee, ankle, hip, shoulder

^a^The presence of intra-articular effusion is included in the scanning protocol but owing to its fluctuating nature is not considered in the scoring system. Reproduced with permission from Thieme ^33^.

The authors of this very valuable systematic review ^51^ concluded that there is currently fair evidence (grade B) to recommend MSKUS as an accurate technique for the early diagnosis of HA (with particular regard to soft tissue abnormalities), to demonstrate that MSKUS scores correlate with clinical/ultrasound constructs, and to prove an association between MSKUS findings and functional joint status. However, there is still insufficient evidence (grade I) to conclude that MSKUS-detectable findings are responsive or sensitive to changes in prophylactic therapy in children or adults with HA. The evidence was insufficient in both quality and quantity.

## A new wave in the evaluation of hemophilic arthropathy

Over the last decade, POC-MSKUS has emerged as a critical tool in the evaluation of hemarthrosis and painful musculoskeletal episodes in PwH ^32, 37– 40, 66, 67^. One group, Ceponis *et al*., demonstrated that hemarthrosis was present in only one third of acute painful joints ^38^ and that disappointingly about two thirds of painful musculoskeletal episodes were judged incorrectly by either the patient or physician. POC-MSKUS findings changed the management in more than 70% of episodes, which resulted in symptom improvement in more than two thirds of the cases. Wider use of POC-MSKUS may serve to substantiate these data. Furthermore, with appropriate training, POC-MSKUS has been increasingly used as part of the bedside evaluation of muscle hematomas in PwH ^68, 69^ and for ultrasound-guided joint aspiration and intra-articular corticosteroid injection for symptomatic relief and pain control in PwH and chronic joint pain secondary to advanced HA ^70, 71^. A very recent article by De la Corte-Rodríguez *et al*. showed that arthrocentesis carried out immediately after the diagnosis of acute hemarthrosis in strictly aseptic conditions and under hemostatic coverage was well tolerated and accelerated joint recovery ^72^. Lastly, Rezende *et al*. demonstrated that joint lavage followed by injections of triamcinolone and Hylan G-F 20 (viscosupplement) improved balance, function, and bleeding events in patients with severe HA ^73^, and Li *et al*. suggest that a single intra-articular platelet-rich plasma injection results in better improvement in pain relief, joint function, and reduced synovial hyperemia when compared with five weekly intra-articular injections of hyaluronic acid in patients with HA of the knee ^74^.

Since the systematic review by Ligocki *et al*. ^51^, the “Joint Activity and Damage Exam” (JADE) POC scanning and scoring protocol has been developed and validated by Volland *et al*. ^75^ as the first quantitative POC-MSKUS algorithm for precise measurements in hemophilic joints of adult PwH. The protocol evaluates for effusions and measures osteochondral surface defects, cartilage thickness, soft tissue expansion, and microvascular perfusion abnormalities on PD for ankles, elbows, and knees. Validation studies showed high intra-/inter-rater reliability, which the authors hope would allow easy quantification of the progression of HA. To date, a similar quantitative POC-MSKUS protocol for children with hemophilia has not been proposed, and the semi-quantitative Hemophilia Early Arthropathy Detection with UltraSound (HEAD-US) POC protocol of Martinoli *et al*. ^64^ remains the most widely used in this patient population for the early detection of HA ^45, 64, 76^. The protocol has an additive score to a maximum score of 8 for synovial hypertrophy and cartilage and bony changes. Joint effusions are excluded from the HEAD-US score, and PD imaging is not carried out as part of the scanning protocol; validation studies of the additive HEAD-US score have not yet been published. Although the protocol has its limitations, its few to no measurements, short learning curve, and proposed examination times of less than 2 to 5 minutes per joint are attractive to busy clinicians and physical therapists in the outpatient setting. The authors propose that a six-joint baseline scan of bilateral elbows, knees, and ankles can be completed within 30 minutes and this protocol has demonstrated good reliability when performed by clinicians and non-radiologists after only limited training ^77– 79^. Furthermore, HEAD-US scores correlated well with HJHS for the elbows, knees, and ankles, and the correlation coefficient was as high as r = 0.70 ( *P* <0.01) in one study ^56^. In a more recent series by De la Corte-Rodríguez *et al*. ^80^, 14% of patients exhibited HEAD-US signs of early HA in joints without reported bleeds and an HJHS 2.1 score of 0. The authors concluded that, in their experience, the HEAD-US scanning and scoring protocol was superior to bleeding history and HJHS 2.1 score in the early detection of HA. The right ankle was the most severely involved joint in this report. These findings were supported by several other studies highlighting the sensitivity and utility of HEAD-US score in the early detection of HA as compared with HJHS or physical exam alone ^64, 81– 83^.

The diagnostic accuracy of the HEAD-US, POC-MSKUS protocol for the detection of HA biomarkers, compared with joint MRI, was recently evaluated by Foppen *et al*. ^84^. HEAD-US was found to be able to accurately assess the presence/absence of synovial hypertrophy in joints of PwH with a positive predictive value of 94% (confidence interval (CI) 73–100%) and a negative predictive value of 97% (CI 91–100%). The HEAD-US and IPSG MRI scores had a strong correlation of r = 0.90 ( *P* <0.01) for the severity of synovial hypertrophy as well. These findings are very promising since synovial hypertrophy is associated with an increased risk of bleeding ^85^ and may be responsive to treatment ^86^; early detection with POC-MSKUS may allow customization and personalization of treatment regimens for PwH in the clinic as they actively participate in the scanning process and review findings in “real time” with their clinical providers.

The overall diagnostic accuracy of HEAD-US for synovial hypertrophy detection was 97% but was not as high for osteochondral surface abnormalities and this is similar to what was described for full-joint MSKUS in the past ^41, 42^. Limited penetration power of the ultrasound beam means that most of it is reflected over the bony surfaces and the evaluation is limited primarily to peripheral joint surfaces; POC-MSKUS can provide only a general idea of the osteochondral surface and whether cartilage and bony alterations are present or absent. MRI is still the best imaging modality for the detailed evaluation of osteochondral derangements in HA, particularly those pertaining to the central aspect of the cartilage and subchondral bone evaluation ^33, 41, 61, 65, 87^.

## Gaps in knowledge and Future directions

POC-MSKUS promises to improve our workflow, eliminating the need for sedation in children and allowing the early detection of HA in its subclinical stage; several gaps in knowledge continue to exist and questions remain unanswered. A number of scanning/scoring protocols ^10, 31, 33, 41, 45, 53, 60, 75^ have become available, but they have not been globally standardized or validated, limiting their widespread use and implementation for that purpose. To achieve this goal, we need to reach a consensus regarding which sonographic biomarkers of HA should be included in the POC-MSKUS score(s) and what the POC-MSKUS definitions of pathologic conditions are 88. POC-MSKUS and full-joint MSKUS scanning and scoring protocols should be compared, standardized, and evaluated against the reference standard (MRI) before they are globalized. As POC-MSKUS has long been used in rheumatology, following Outcome Measures in Rheumatology (OMERACT) guidelines when developing new imaging modalities for arthritic conditions would prove beneficial ^88– 90^. Well-designed longitudinal cohort studies to evaluate the value of ultrasound in the early detection of HA and its clinical impact on treatment strategies and prophylactic regimens are urgently needed ^51^, as are guidelines and recommendations for necessary training and appropriate use and implementation of POC-MSKUS as a diagnostic tool in PwH ^91^.

The highly operator-dependent nature of ultrasound justifies the initial skepticism it was met with and necessitates rigorous operator training to avoid misuse and misdiagnosis ^69, 91^. Currently, formal certification in MSKUS scanning is not required to implement POC-MSKUS in clinical practice, but resources are available and could be used to achieve this end. The need for additional and/or special training in POC-MSKUS scanning becomes evident when evaluating pediatric patients, muscle injuries, and other joints (hips and shoulders). When a pediatric joint is scanned, a sophisticated understanding of the immature skeleton, appearance of growth plates, secondary ossification centers, and areas with abundant normal periarticular fat is key ^65^. The paucity of available literature coupled with the need for specialized training and challenges in image acquisition in the more active and often less cooperative pediatric patient contributed to the underutilization of this technique for assessing pediatric joints in both research and clinical practice. Atlases on expected soft tissue and epiphyseal cartilage thickness and vascularization, and normal ossification of the joints of maturing healthy children are needed ^92– 95^. Awareness of normal variation and contralateral comparison with the other joint, if normal, are helpful. If POC-MSKUS does not provide an answer, referral for a full-joint MSKUS or other imaging modalities such as MRI and plain x-ray should be made ^65, 87, 91^.

Additionally, it is important to note that although MSKUS can readily distinguish between fluid and soft tissue ^10, 32, 37, 38, 43^, MSKUS remains less sensitive in soft tissue discrimination (fat versus synovium) and in detecting hemosiderin, which remains debatable and controversial ^49, 96^; some studies describe distinctive echotextural features distinguishing hemosiderin from synovium ^10, 41, 61^ and others do not find a difference in the sonographic appearance of hemosiderin-laden and hemosiderin-free synovium ^96, 97^. MSKUS unreliability/inability to detect hemosiderin deposition in the synovium of PwH will compromise its ability to detect “microbleeds”, an almost decade-old concept that refers to asymptomatic or subclinical leakage of microscopic amounts of blood into a joint hypothesized to contribute to deterioration of the joint without clinical evidence of hemarthrosis ^2, 44, 56, 80, 81, 98^. Although we do not currently have direct evidence that joint microbleeding occurs in PwH, this does not mean that it does not exist! In a very recent review, Puetz ^98^ sheds some light on this topic and argues that medical imaging findings in joints free of clinically evident bleeds and symptoms (MIFFS) constitute a significant clinical issue that deserves appropriate investigation; assuming that MIFFS in PwH are directly related to or caused by microbleeds (or vice versa) can lead to unnecessary, or ineffective medical treatment.

Lastly, the value of three-dimensional ultrasound and the newer advances in ultrasound technology in the evaluation of HA has yet to be determined. Currently, most scoring protocols are semi-quantitative. Do we foresee a future where artificial intelligence algorithms automate the quantification and assessment of soft tissue proliferation, cartilage thickness loss, degree of bony derangement, and hypervascularity making the process less operator-dependent and time-consuming?

## Closing remarks

Continued innovative development and widespread utilization of POC-MSKUS are expected over the next few years. The dynamic, timely, beneath-the-surface evaluation of hemophilic joints at the bedside using POC-MSKUS as an adjunct to physical examination is invaluable and ultimately will not only result in a more refined understanding of the pathophysiology of HA and natural course of the disease but also enhance patient/parent compliance and adherence to treatment regimens for both acute injuries and prophylaxis. POC-MSKUS can provide visual evidence of joint health which can be used as a motivational tool for prophylaxis-reluctant or non-adherent patients at visits to their hemophilia treatment centers ^4, 99– 101^.

In the recently published IPSG survey of the use of ultrasound for assessment of musculoskeletal disease in PwH ^102^, POC-MSKUS was most often performed by a physiotherapist (53%) or a hematologist (43%) and was perceived as most useful to confirm an acute joint bleed and the presence of synovial hypertrophy. The greatest perceived barriers to the implementation were lack of trained health-care professionals and the overall time commitment required to perform the exam ^102^.

Recently, Zhou *et al*. explored the potential of a pocket-sized handheld ultrasound device for the evaluation of joint effusions and hemarthrosis in hemophilia ^103^. The image quality of the handheld ultrasound device was deemed sufficient to identify joint landmarks necessary to accurately localize effusions, and the device was promising to be both feasible and reliable even when performed by non-experts (such as medical students) after a brief training period ^103^. Perhaps it is possible to envision a future where patients are equipped with handheld ultrasound devices, but important questions remain to be resolved. Would this allow more efficient use of factor products and improved adherence to prophylactic regimens or false reassurance by false-negative scans and subsequent under-treatment? Will POC-MSKUS finally provide us with an objective rather than subjective annualized bleeding rate, allowing a more accurate comparison of the hemostatic effect of different factor products and newer non-factor therapies, or will it leave our patients feeling unheard? Is POC-MSKUS going to fuel the microbleed debate? Can POC-MSKUS accurately detect microbleeding and differentiate acute on top of chronic bleeding episodes in persistently hemorrhagic target joints? Where do POC-MSKUS and pocket handheld devices fit into personalized treatment strategies and outcome measures ^104, 105^? Finally, will ultrasounds bridge the physical distance between patients and their hemophilia treatment centers, allowing them to have a more objective evaluation of their acute musculoskeletal painful episodes remotely, or will it result in non-compliance with poor follow-up when the home scans reassure against progressive joint disease and the perceived role of the clinical exam is lost?

POC-MSKUS is very promising, yet we should acknowledge the need for formal training, attaining and maintaining competence, quality assurance, and understanding its appropriate use ^91^. International consensus guidelines and recommendations for use should be established to allow the effective implementation and utilization of this imaging modality in hemophilia. Studies to evaluate the feasibility of implementation and time needed to complete scans for both the evaluation of acute musculoskeletal concerns and longitudinal joint disease monitoring are needed. Frequency of six-joint surveillance ultrasound needs to be established for asymptomatic, near-asymptomatic, and target joints. Objective evidence highlighting the limitations of POC-MSKUS should be provided. What POC-MSKUS can and cannot do should be clearly disclosed to patients/parents, and when the question posed is not answered, the referral to a diagnostic imaging department and an experienced musculoskeletal radiologist for either a full-joint MSKUS or a different imaging modality should be made. As beautifully stated by Lawson *et al*., “point-of-care ultrasound should be respected for the complexity of the technology and the skill required to interpret it” ^91^. Only then will we avoid misdiagnosis and misuse.
